# Disclosing neuroimaging incidental findings: a qualitative thematic analysis of health literacy challenges

**DOI:** 10.1186/s12910-016-0141-1

**Published:** 2016-10-11

**Authors:** Caitlin E. Rancher, Jody M. Shoemaker, Linda E. Petree, Mark Holdsworth, John P. Phillips, Deborah L. Helitzer

**Affiliations:** 1 The Mind Research Network, Albuquerque, NM 87106 USA; 2 College of Pharmacy, The University of New Mexico Health Science Center, Albuquerque, NM 87106 USA; 3 Department of Neurology, The University of New Mexico, Albuquerque, NM 87106 USA; 4 College of Population Health, MSC 09-5070 Health Sciences Center, University of New Mexico, Albuquerque, NM 87131-0001 USA

**Keywords:** Health literacy, Incidental findings, Neuroimaging

## Abstract

**Background:**

Returning neuroimaging incidental findings (IF) may create a challenge to research participants’ health literacy skills as they must interpret and make appropriate healthcare decisions based on complex radiology jargon. Disclosing IF can therefore present difficulties for participants, research institutions and the healthcare system. The purpose of this study was to identify the extent of the health literacy challenges encountered when returning neuroimaging IF. We report on findings from a retrospective survey and focus group sessions with major stakeholders involved in disclosing IF.

**Methods:**

We surveyed participants who had received a radiology report from a research study and conducted focus groups with participants, parents of child participants, Institutional Review Board (IRB) members, investigators and physicians. Qualitative thematic analyses were conducted using standard group-coding procedures and descriptive summaries of health literacy scores and radiology report outcomes are examined.

**Results:**

Although participants reported high health literacy skills (m = 87.3 on a scale of 1–100), 67 % did not seek medical care when recommended to do so; and many participants in the focus groups disclosed they could not understand the findings described in their report. Despite their lack of understanding, participants desire to have information about their radiology results, and the investigators feel ethically inclined to return findings.

**Conclusions:**

The language in clinically useful radiology reports can create a challenge for participants’ health literacy skills and has the potential to negatively impact the healthcare system and investigators conducting imaging research. Radiology reports need accompanying resources that explain findings in lay language, which can help reduce the challenge caused by the need to communicate incidental findings.

## Background

Advancements in Magnetic Resonance Imaging (MRI) technology allow researchers to more accurately detect individual differences in neuroimaging scans. Findings of potential health or reproductive importance for the individual research participant, which are outside of the aims of a broader research study, are known as incidental findings (IF) [1]. These findings are detected in up to 70 % of MRI scans. However, investigators have concerns about providing research MRI findings to participants as they may contribute to participant distress or anxiety, unnecessary medical appointments, and financial burden as well as risks of institutional liability, ramifications for future recruitment and overtaxing the healthcare system [2–9]. Regardless, previous reports consistently show that research participants want to know about incidental findings found on their MRI scans [10–13].

The Presidential Commission for the Study of Bioethical Issues offers some guidance on developing IF management processes [14, 15]. Investigators are encouraged to anticipate the possibility of encountering IF on MRI scans; yet the report does not offer guidance on which, if any, findings should be returned to research participants or how to disclose the information. Our imaging center incorporates this guidance and in the development of our own IF management process, we have implemented a system to review all MRI scans (per local IRB mandate) and provide full disclosure of all findings to research participants. (For a full description of the disclosure system see [15, 16]). As per institutional policy, all research participants involved in an MRI study receive a radiology report with a descriptive summary of any findings written by a board-certified radiologist and a recommendation on whether they should follow up with a physician based on the findings. While the system has adapted to a current binary recommendation rating system of “Please see your doctor about this report” or “You do not need to see your doctor about this report,” until very recently research participants received one of the original 5-point rating system recommendations: “No Abnormal Findings,” “No Referral Necessary,” “Routine Referral,” “Urgent Referral,” or “Immediate Referral.” Those participants who received a rating of “Routine,” “Urgent,” or “Immediate” referrals were advised to seek follow-up care with their physician. All reviews indicate, regardless of the radiologist findings and referral recommendation, if a participant is experiencing symptoms they should follow up with their physician. A detailed description of the review process, information received by the radiologist and how these referrals are categorized is available elsewhere [15]. One of the difficulties of this system was the complex nature of the radiology report returned to participants.

The challenge for individuals to make appropriate decisions from complex health information is a national problem that extends beyond neuroimaging. Recent federal policy initiatives, including the Affordable Care Act, the Department of Health and Human Services’ National Action Plan to Improve Health Literacy and the Plain Writing Act each call attention to health literacy’s major role in improving healthcare and health for all Americans [17]. Most healthcare materials continue to be written above the 10th grade reading level [18, 19], despite (a) the overwhelming evidence that poor health literacy leads to medication errors, higher hospitalization rates, and lower use of preventive services; (b) new policy from major regulatory bodies (e.g., the Joint Commission’s efforts to make effective communication an organizational priority); and, (c) relevant to this study, radiologists’ calls to improve information content [20–26].

The purpose of this study was to identify the extent of the health literacy challenges created when returning incidental finding reports to neuroimaging research participants. We report on findings from a retrospective survey and focus group sessions with major stakeholders involved in disclosing IF. Our study had two main aims: first, to explore the health literacy abilities of former research participants and characterize their experience receiving IF information; and second, to measure the broader impact of returning IF information on neuroimaging investigators, Institutional Review Board (IRB) members and primary care physicians.

## Methods

Full descriptions of methods and subject demographics for both the retrospective survey and focus group study have been published elsewhere [13, 15, 27]. The study was approved by the University of New Mexico Human Research Review Committee. We conducted a systematic survey of the perceptions and preferences of three key stakeholder groups: former research participants, Institutional Review Board (IRB) members associated with the University of New Mexico (UNM), and investigators conducting MRI studies [13]. Only results from the former research participant surveys are reported here.

We recruited participants for the focus groups using a purposive sampling approach. We identified six key stakeholder groups (prior MRI research participants, parents of children who were MRI research participants, community members, IRB members, investigators associated with our imaging center and physicians) to participate in focus group discussions at our active neuroimaging research facility. We identified prior research participants and parents of child research participants from an existing list of potential subjects who indicated their willingness to be contacted for future research. Trained staff utilized three main criteria to assure desired balanced participant demographics: sex, age, and ethnicity. We randomly selected potential participants from the contact list until these cells were balanced. Once identified, we conducted additional eligibility screening over the phone which included: 1) remembering receiving their MRI scan results from a research study; 2) confirming a willingness to talk about their experience in MRI research with a group of unfamiliar peers; and 3) demonstrating the ability to communicate in English. We conducted a total of three participant and four parent focus groups. We recruited community member participants through internet advertisements (e.g., Craig’s List) and required them to meet three eligibility criteria: 1) willingness to talk with a group of unfamiliar peers; 2) the ability to communicate in English; and 3) research naiveté, such that they had no prior experience participating in a research study. We employed a similar stratified approach to assign community members to balanced demographic groups. We conducted a total of three focus groups with community members. Research staff identified IRB Members and investigators via publically available rosters and by attending scheduled staff meetings, followed by brief email solicitations. We conducted a total of three IRB member and three investigator focus groups. Similarly, we recruited Primary Care physicians at both Family and Community Medicine and Internal Medicine department faculty meetings as well as through email solicitation. We completed a total of two physician focus groups. Focus group discussion guides for all subjects are available elsewhere see, [27].

All subjects provided verbal consent for study participation. We analyzed transcripts using principles of Grounded Theory and group consensus coding [15]. All participants who were re-contacted to take part in the study had previously received a formal radiology report of their research MRI scan, as per institutional policy. We assessed health literacy in both the survey and focus groups using A Brief Health Literacy Tool [28]; the retrospective survey participants completed the 4-question assessment on a slider scale with averaged scores ranging 1–100, while focus group subjects completed the assessment using the original 5-Point Likert Scale with final scores ranging 0–16. We transformed the focus group scores into the 1–100 range scale for comparative analysis. We conducted qualitative thematic analyses using standard group-coding procedures and descriptive summaries of health literacy scores. We also examined radiology report outcomes. A total of *N* = 196 research participants completed the retrospective survey and *N* = 151 subjects (25 research participants, 23 parents, 27 IRB members, 29 investigators, 17 physicians and 30 community members) participated in 16 focus group discussions.

## Results

### Health literacy scores

**Fig. 1:**
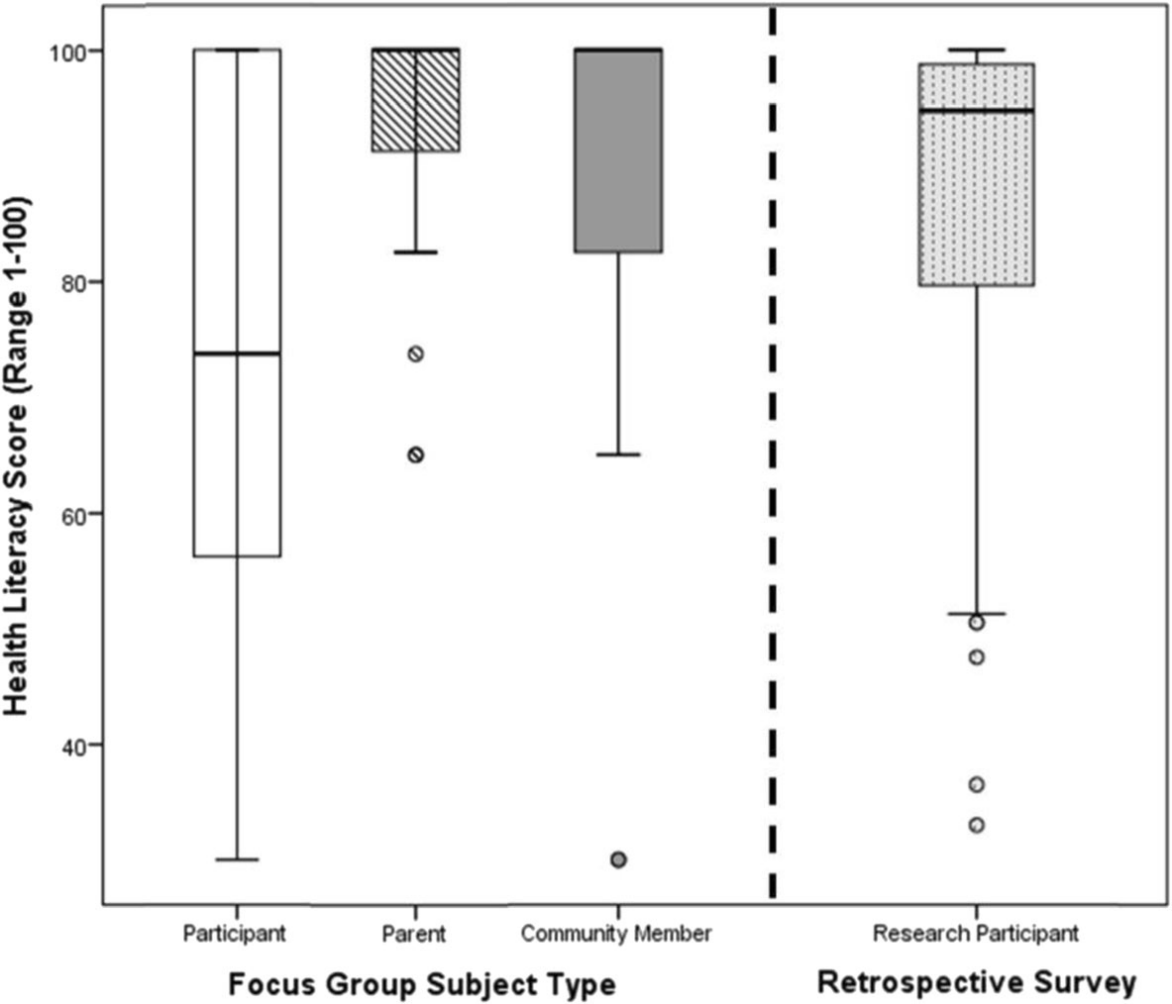
Health literacy scores by subject type. Box-plot comparisons of subjects’ health literacy scores based on composite scores from a standard measure ranging from 1 to 100, with higher scores signifying higher levels of health literacy. Analysis of variance statistic reports a significant difference across focus group subject type, *p* = .002

Research participants in the retrospective survey reported high average health literacy scores, mean = 87.3, SD = 14.9. Similarly, the participants, parents and community members who completed the health literacy measure in the focus group discussions reported high health literacy: research participants m = 82.3, SD = 15.5; parents m = 94.8, SD = 8.1; and community members m = 92.5, SD = 13.3. The health literacy scores between the focus group subjects were significantly different with *p* = .002 (see Fig. 1).

**Fig. 2:**
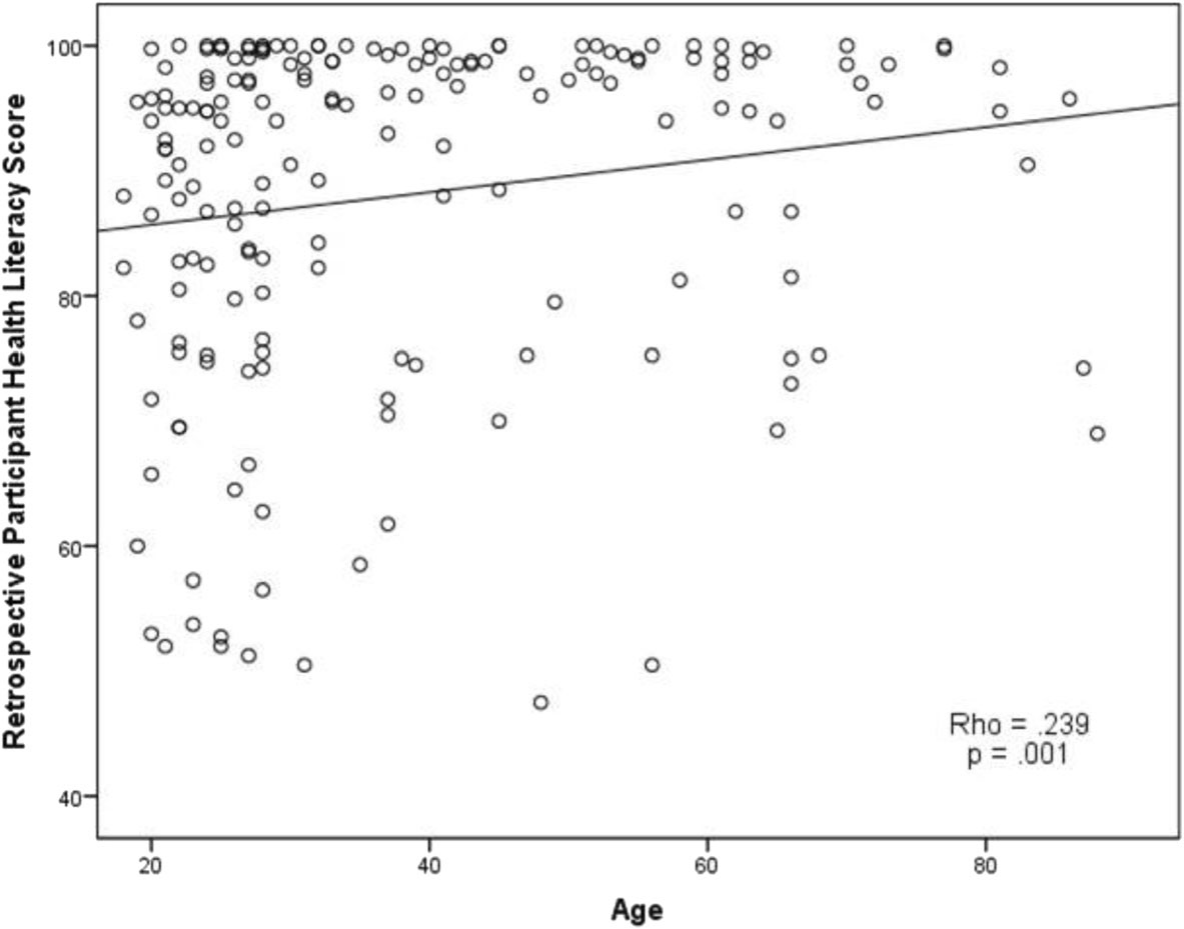
Retrospective participant health literacy score by participant age. Plotted distribution of research participant health literacy score based on a composite score from a standard measure ranging from 1 to 100, with higher scores signifying higher levels of health literacy. A significant positive association exists between health literacy score and participant age, *p* = .001

Previously we have reported that health literacy scores for retrospective survey participants were negatively correlated with baseline health anxiety scores (*p* = .01) and participant anxiety level in response to receiving the radiology report (*p* = .002) [13]. Participant health literacy was also positively correlated with participant age (*p* = .001, see Fig. 2).

### Research participant healthcare utilization

Of the *N* = 196 participants who completed the retrospective survey and received a radiology report with a radiologist summary and recommendation: 38 % received “No Abnormal Findings,” 41 % “No Referral Necessary” and 21 % “Routine Referral.” Despite the referral recommendations 9 % (*n* = 7) of participants who received “No Referral Necessary” sought further medical evaluation with their physician concerning their MRI scan results; conversely, 67 % (*n* = 26) of participants who received a “Routine Referral” did not seek any kind of follow up medical care.

A similar trend was noted in the focus group discussions with research participants and parents of child participants. We gave all focus group participants an example of a radiology report with a finding that stated “No Referral Necessary.” In response to this recommendation a strong theme emerged amongst the research participants that, despite being told otherwise, they would still seek to follow up with a medical professional. As one participant stated:

*“It says you don’t need to see a doctor, but that doesn’t mean you can’t see a doctor.” (participant)*

This theme arose more frequently in the parent focus group discussions as they considered the health of their child. When discussing their experience receiving the radiology report from their child’s MRI study some parents explained:

*“There’s something in his brain! You can’t just sweep that under the rug. I mean, obviously they found something and they they’re saying 'do not see a doctor'. They’re trying to let you know not to make a big deal about it, but then yet this is your kid and they see that they found something wrong. How can you just ignore that?” (parent)*

*“My letter just said that they found a cyst […] and it said you do not need a follow up, but it did say they found a cyst. I was thinking, how do they not want me to follow up and they’re telling me he has a cyst, how do I not follow up on this?” (parent)*

*“Now I could look at this and probably look some stuff up and have some questions that when I go to my primary and say, ‘Yea. They told me I don’t really need to talk to you about this, but I have some concerns.’” (parent)*

*“Oh my god. I’d be going [to my child’s doctor] even though it says not to.” (parent)*

### Qualitative responses to radiology report

#### Retrospective survey comments

We gave retrospective survey participants the opportunity to comment on their experience in receiving the radiology report following their MRI scan. Many participants reported they did not understand the content of the radiologist summary within their MRI results. As one participant stated,

*“My MRI results: ‘Scattered white matter bright areas involving the periventricular/subcortical white matter. May represent microvascular ischemic change.’ Useful information to another radiologist, but not to me.” (participant)*

Research participants expressed a desire to have greater clarification on the radiologist summary. To obtain information participants reported seeking out explanations through the internet or by asking their physician. Two participants commented:

*“The single line statement given to me I had to look up online to understand what it meant, if anything,” (participant)*

*“Results were unclear until I discussed with primary physician [sic].” (participant)*

#### Focus group comments

Two salient themes emerged when focus group research participants and parents of subjects discussed what their responses would be to receiving a radiology report such as the one provided in the focus groups. First, all subjects felt it was difficult to understand the radiology report because it was written in medical terminology. As one research participant commented:

*“There’s no way to really tell a lot of times if you have a sinus infection or if you have brain cancer. There’s just jargon on there in my eyes, it’s just whatever the radiologist and physician want to put on there. It means nothing to people like me.” (participant)*

This sentiment was echoed throughout the focus group discussions – research participants and parents of participants discussed how incomprehensible they found the language of the radiology report. Participants reiterated this common theme:

*“I’m not a doctor, so I have no idea what that means.” (participant)*

*“Bunch of words that I don’t know and some numbers here and there, and pi this and you know, it’s hard to put together what it is.” (participant)*

*“I look at the doctor’s report, but then well I don’t really understand that fully.” (participant)*

*“The reports that I get you know a radiologist could read it, a physician could read it, a student could probably read it in medical school, but in layman’s terms it would be nice to find out what the jargon is.” (participant)*

*“It was kind of vague, we went through the same experience where they were like, ‘So, what do you want to do with this? I don’t even know what it is.’” (parent)*

*“I didn’t understand anything they had wrote on the paper [sic].” (parent)*

*“It made it even a little more confusing though because they were like ‘well there’s a bright spot.’ Is there a light in her head? What’s the deal?” (parent)*

Another consistent theme throughout the discussion on the radiology report was that both research participants and parents of participants desire to receive additional information explaining their IF alongside the radiologist summary. Representative comments from these discussions follow:

*“I wish that information in more general terms was on my copy, front page, so that when I look at the radiologist’s summary that says may or may not be of medical concern, right below it I’m not freakin’ out because I’m like oh look, general terms, I’m okay, common that I have a cyst, not a big deal [sic].” (participant)*

*“If they’re specific about what you need to do afterwards, like if you have this kind of problem then you need to see this kind of specialist in this kind of time frame. And then what kind of risks are involved with it, you know what I mean? … As detailed as possible.” (participant)*

*“We would like to see numbers or a graph or something. If I saw that my kid was maybe like ¾ above the level, compared to other kids, then I would have been like, ‘Okay. Well, that’s not that bad.’” (parent)*

*“I really like the idea if they listed links of the bottom or something where you could go and specifically call or talk with someone that you could ask some specific questions.” (parent)*

### Professional perspective on health literacy burden

The concept of radiology reports presenting a health literacy burden was also prevalent in the focus group discussions with current investigators, physicians and IRB members. These stakeholders similarly agreed that the radiologist summary is not accessible to a lay population.

*“The report is just ‘hey, the kid has a cold so their sinuses are clogged.’ But instead of saying it like that I can’t even come up with what they’ve said. And so then the parents call you up and you’re like, ‘oh your kid has a stuffed nose.’”* (investigator)

*“I think sometimes it’s just the language that’s used is confusing.” (investigator)*

*“[Participants] may have the cognitive capacity to consent for the minimal risk study, but again, they may not understand the actual impact of what the findings may mean.” (investigator)*

*“[We physicians] have the experience and knowledge to understand the worthlessness of knowing a lot of those things and some of the general study participants would have a harder time with that.” (physician)*

*“I think it could be potentially damaging if you tell somebody all of the things you found that were maybe benign and then they go out and commit suicide because they don’t know or didn’t get the correct follow up for those things, or weren’t told about them in great detail.” (IRB member)*

A theme unique to the IRB member discussion was the potential ethical dilemma of returning radiology report information that participants cannot understand. IRB members suggested that IF reports should be as understandable as consent forms, which are written at an appropriate level for all potential research participants. As one IRB member stated:

*“[Consent forms] are usually full of jargon, they’re not written with the participant in mind. They’re written with the lawyers or other investigators in mind. So to me how [radiology reports] are phrased and who’s gonna do the verbal piece around it and how familiar are they with that population, particularly if it’s a vulnerable population, would be a real issue for me as a reviewer.” (IRB member)*

Investigators and physicians spoke about the burden of managing participant responses to their radiology reports. Investigators specifically discussed the impact on their research time and future participant recruitment.

*“We have a very sensitive population that we work with and one of our parents received a report and it wasn’t anything significant but it was an incidental finding […] and they freaked and they called their case manager and they talked to other parents and so now everybody is like, ‘Well, what about this person? They found something. What’s wrong with their kid? Did you guys do that? Did the MRI do that?’ So there’s a lot of that that we deal with sometimes.” (investigator)*

*“We’ve had a complaint that too many non-significant findings were burdening people that we were putting the responsibility on to deal with these reports.” (investigator)*

*“We get a lot of people calling us up to have our scans sent to doctors, for things that are pretty normal.” (investigator)*

*“It could get very expensive and inconvenient.” (physician)*

*“I just worry… I have so many patients who want to come for appointments and can’t get in and I worry about six more appointments wasted to deal with this kind of thing.” (physician)*

The physicians in focus groups discussed the potential burden to the healthcare system if their patients followed up on reports they received from a research study describing medically insignificant IF. In response to a potential case study of a patient receiving a radiology report from a research study with a recommendation of “No Referral Necessary,” but still seeking clinical guidance, the physicians’ overarching perspective can be summarized as follows:

*“There’s nothing here to treat. It’s not gonna change how you deal with your patient. You’re gonna have to educate her, you’re gonna have to work with her, you’re gonna have to have her come back in for annual visits and checkups, but that’s not gonna change anything. It will give her an anxiety disorder [sic].”* (*physician*)

## Discussion

All stakeholders involved in disclosure of incidental findings from MRI research have significant concerns regarding the incomprehensible medical jargon in radiology reports; however, the challenge for investigators and radiologists is that simplifying the radiology report information alone would reduce its clinical utility for follow up care. Incidental finding reports written by radiologists in complex language may diminish the autonomy of participants with low health literacy skills. Autonomy is traditionally considered a principle underlying informed consent. In order to optimize participant autonomy, there are ethical principles that support the provision of unexpected information to participants, including IF [29]. In the context of returning radiology reports, autonomy requires that investigators grant individuals sufficient information to make educated decisions regarding their follow up care. However meeting this autonomy principal is challenging for investigators when their research participants have low health literacy skills. Investigators could consider reconfiguring their approach to reporting incidental findings by focusing on those participants with the most limited health literacy [30]. Doing this would require ongoing measurement of health literacy levels. Absent this measure of health literacy abilities, the most ethical approach is to offer all participants their research findings in comprehensible language, or at an 8th grade reading level [30]. However the difficulty is how to provide this information while still retaining the critical clinical specificity required for any necessary follow-up.

There is consensus among all stakeholders in our study that the only approach is the ethical approach: IF need to be reported to participants. This finding is comparable to the existing literature describing participant’s expectations for participating in research studies [10–13]. In work by Kirschen and colleagues, over 90 % of their research participants wanted IF information communicated to them [10]. This demand for information was recently echoed in the Presidential Comission for the Study of Bioethical Issues’ report for investigators to anticipate and communicate their approach in managing IF [14].

Our own empirical work reveals that the complexity of the information in research radiology reports contributes to the challenges of making appropriate follow up decisions. Despite very clear recommendations of whether or not to seek medical care, 10 % of participants in our preliminary research sought follow up care without referral and 67 % did not seek medical care when directed to do so. Similar to the retrospective survey participants, the parents of child participants who took part in the focus groups shared that if they do not understand information pertinent to their child’s health, they will seek medical evaluation – regardless of the report’s recommendation that follow-up medical care is unnecessary. These findings suggest participants may be making inappropriate healthcare decisions based on their inability to understand their research radiology report. Measuring broad outcomes in literature concerning health literacy suggests poor health literacy skills are detrimental to patient health in many ways, including inappropriate use of medical services [21–23]. It is important to note that our survey did not query participants about whether they already knew about the finding listed in their radiology report, so it is possible some participants did not seek follow up care for findings about which they and their physician were already aware. Still the potential consequences of misunderstanding the information in the research radiology report puts a considerable burden on investigators to communicate that information clearly and accurately.

In this manner, participant’s health literacy skills present a challenge for investigators to determine the optimal disclosure of IF information. Even as participants reported they did not understand the radiologist summary, they also reported high health literacy skills. Within the retrospective survey, those participants who reported having higher health literacy were likely to be older and less anxious about their health. These skills do not necessarily reflect an ability to successfully interpret complex radiology review information. Having self-confidence (equated with high health literacy) in their health navigation skills or in their ability to complete medical forms does not indicate an ability to dissect neuroimaging jargon (nor should that be expected of them).

Furthermore the results of our study highlight one of the challenges in measuring participant health literacy using brief instruments – such as the Chew instrument we used in our surveys [28]. Researchers have several choices: administer a face-to-face instrument or rely on participants’ abilities to independently complete a self-administered measure. Further, within each type, researchers have to choose between a briefer health literacy measure that may provide more limited and potentially misleading information, or a longer instrument that provides more information but may increase participant burden. A shorter measure, such as the Chew instrument, asks participants to rate their self-confidence to perform a variety of health care tasks like “completing medical forms” or “reading the instructions on a prescription bottle.” While the Chew questions address participant self-efficacy beliefs regarding their health management skills, this instrument falls short of the more in-depth, face-to-face instruments such as the Test of Functional Health Literacy in Adults (TOFHLA), that measure tangible health literacy skills such as interpreting the instructions on the label of a bottle of Tylenol [31]. In the context of neuroimaging and returning IF information, inaccurate measurement of health literacy leads to the conclusion that investigators must design radiology reports to meet the needs of individuals, whose health literacy skills can vary widely.

A consistent theme from participants who participated in the focus groups was their desire to have the ability to follow up with someone, to ask questions or to receive more insight to help explain their findings. This finding is similar to other measures of research participant expectations from receiving IF – participants desire someone they see as reputable to help explain the reports [10–13]. In a preliminary effort to address this issue, our research institution includes a cover letter accompanying all radiology reports that explained the general nature of IF in lay language, listed the contact information for our Medical Director and invited participants to contact the him in the event that they had any questions about their radiology report or would like to discuss their findings. Unfortunately the feedback we received from participants in the retrospective survey and the focus groups leads us to believe that the cover letter was not maximally effective in communicating how and from whom participants could seek additional information about their IF.

We would advocate for materials designed for participants with the lowest level of health literacy skills and the use of other delivery mechanisms to ensure the information is accessible to all individuals. The health literacy literature advocates that all information should be written at the 8th grade level or lower, despite whether the population is college educated or not.

The discussion in the focus groups demonstrates the concern physicians have about the burden health literacy challenges can have on the healthcare system. Physicians expressed they did not have the time to address patient anxieties raised by research reports about benign IF. Physicians feel stretched to meet their current patient loads; the imposition of additional medical appointments for this purpose could further burden an already overtaxed system. To date, our research locally suggests the potential workload for the healthcare system caused by reporting IF is minimal [15], but in general, participants with low health literacy skills represent a constant challenge for the healthcare system. From the participant perspective, it is well documented that health literacy affects health outcomes [18–23, 27–30]. The health system should address the broader challenge of managing effective patient education and targeting health communications to the needs of those with limited health literacy.

Similar to the challenges expressed by physicians in the healthcare system, investigators must manage the potential research burdens created by participants’ inability to understand their radiology reports. First, there is a cost to investigators when time must be allocated to explain the findings to individual participants. This reflects some concern in the existing literature regarding the existing logistical, and financial, cost to managing participant’s IF reports [5]. However, unique to our imaging center, where the investigators in our study perform MRI research, is the cost-effective and logistically stream-lined process of radiology review and report (for full details see, [15]). Within this system, there is already a process where investigators can refer participants to talk to the Medical Director, a certified neurologist, to help explain their findings. Also, investigators fear that one participant’s negative experience with a radiology report might deter other participants from signing up for future studies. This concern is also noted in the literature, as investigators predict receiving ambiguous IF information would cause participants unnecessary fear and anxiety [3–8]. If research participants know each other, or studies are recruiting from a specialized sample, this anxiety may be shared with other prospective participants. However experience shows this rarely occurs; other research finds that 90 % of participants believe receiving IF is a benefit to study participation [13]. Still, investigators fear these types of challenges when providing reports to participants. For this reason, many research facilities do not return findings or only return findings that require immediate clinical follow-up, even though they have been encouraged to do so by recent federal policy initiatives [17].

Until recently, the topic of health literacy has not been openly discussed as a concern of radiologists or investigators who return IF. Our institution’s experience in disclosing IF has revealed the importance of considering a participant’s ability to understand IF information and make appropriate decisions. In order to fully address the burdens created by disclosing complex health information to those with limited health literacy skills, investigators should listen to and learn from participants’ experiences [32]. Investigators face a complex conundrum: they don’t expect participants to understand the radiology jargon, yet they believe that participants ethically deserve to receive the information. Our research shows that participants report a strong desire to receive all medically relevant IF information. Returning radiology reports then, on one hand, creates a burden that investigators and the healthcare system must manage. On the other hand, investigators choose to provide participants with the information to advance patient autonomy. Our research leads us to conclude, as did Volandes & Paasche-Orlow, that disclosure methods should be reoriented to a level that accommodates the needs of participants with the most limited health literacy abilities [29].

Based on participant feedback, our institution modified the recommendation rating scale to a clear binary “Please see your doctor” or “You do not need to see your doctor” in order to meet the needs of those with limited health literacy. Additional investigation is still required to determine whether this relatively new system will result in more appropriate healthcare resource utilization. These findings also indicate a need to establish clear, and descriptive consent language for those research studies that conduct MRI scans and return IF reports. We recommend that investigators use the initial consent conversation to help outline the possible outcomes from an IF report, and direct research participants interested in learning additional information to educational resources. We intend to develop and measure the effectiveness of additional resources (such as an easily navigable website that provides information about the most common brain imaging IF) to enable participants to learn and make appropriate decisions about their specific IF. These informational resources, written in lay language, may address the gap between participant health literacy skills and the complex clinical information provided in the radiology reports, while limiting the amount of burden placed on either investigators or the healthcare system.

This study has several strengths: the diversity of data collection methods, the relatively large sample size, and a participant population that has the experience of receiving IF reports. The study was conducted within one of the few institutions that returns IF reports to every participant. However there are also several limitations to this study. First, the retrospective survey data collected may not accurately reflect current institutional practice. The desire to grant participants in our imaging studies autonomy and improve the accessibility of the reports has resulted in numerous updates to the “radiology review and disclosure” process in a short period of time. Further empirical investigation is still required to fully characterize the burden on the healthcare system and investigators involved in the disclosure process. Second, our desire for a brief health literacy assessment led us to use the Chew measure. However, Chew suggested that the instrument was best used to identify individuals with low to inadequate health literacy and would not be as accurate at estimating distinctions among individuals with higher health literacy [28]. Therefore our ability to accurately assess the range of health literacy among our research subjects was limited. Third, as with all research data emerging from purposive samples, the individuals who participated may not be representative of others who were not included.

## Conclusions

Research participants involved in neuroimaging studies want to receive incidental finding information and want this information to be accessible in language they can understand. The medical jargon in clinically useful radiology reports creates a burden for participants’ health literacy skills and has the potential to negatively impact the healthcare system and investigators who conduct imaging research. To help address these concerns, additional resources accompanying radiology reports are needed to help bridge the communication gap between investigators, radiologists and participants.

## Abbreviations

IF: Incidental findings
IRB: Institutional review board
MRI: Magnetic resonance imaging
MRN: The Mind Research Network
